# Clinicopathological features and prognosis of gastric cancer in young patients

**DOI:** 10.1186/s12885-016-2489-5

**Published:** 2016-07-14

**Authors:** Shushang Liu, Fan Feng, Guanghui Xu, Zhen Liu, Yangzi Tian, Man Guo, Xiao Lian, Lei Cai, Daiming Fan, Hongwei Zhang

**Affiliations:** Department of Digestive Surgery, Xijing Hospital, Fourth Military Medical University, 127 West Changle Road, 710032 Xi’an, Shaanxi China; Department of Dermatology, Xijing Hospital, Fourth Military Medical University, 127 West Changle Road, 710032 Xi’an, Shaanxi China

**Keywords:** Age, Gastric cancer, Young, Clinicopathological features, Prognosis

## Abstract

**Background:**

The clinicopathological features and prognosis of gastric cancer in young patients are both limited and controversial. Therefore, the aim of this study was to define the clinicopathological features and prognosis of gastric cancer in young patients after curative resection.

**Methods:**

From May 2008 to December 2014, 198 young patients (age ≤ 40 years) and 1096 middle-aged patients (55 ≤ age ≤ 64 years) were enrolled in this study. The clinicopathological features and prognosis of gastric cancer in these patients were analyzed.

**Results:**

Compared with middle-aged patients, the proportion of females, lower third tumors, tumor size less than 5 cm, poorly differentiated tumors and T1 tumors were significantly higher in young patients (all *P* < 0.05). The proportions of comorbidity, upper third tumors, well and moderately differentiated tumors, T4 tumors, and positive carcinoembryonic antigen (CEA), alpha fetoprotein (AFP) and carbohydrate antigen (CA) 19–9 were significantly lower in young patients (all *P* < 0.05). The distributions of N status and CA125 were comparable between young and middle-aged patients (all *P* > 0.05). The five-year overall survival rates were comparable between young patients and middle-aged patients (62.8 vs 54.7 %, *P* = 0.307). The tumor location, T status, N status and CA125 were independent predictors of prognosis in young patients. The overall survival of patients with tumors located in the upper or middle third was significantly lower than for those located in the lower third (60.8 vs 50.6 % vs 68.4 %, *P* = 0.016). The overall survival of CA125-positive patients was significantly lower than CA125-negative patients (49.0 vs 64.4 %, *P* = 0.001).

**Conclusion:**

The clinicopathological features were significantly different between young and middle-aged patients. The prognosis of gastric cancer in young patients was equivalent to that of middle-aged patients. Tumor location, T status, N status and CA125 were independent risk factors for prognosis in young patients.

**Electronic supplementary material:**

The online version of this article (doi:10.1186/s12885-016-2489-5) contains supplementary material, which is available to authorized users.

## Background

Gastric cancer is the fourth most common cancer in the world [1] and the second most common cancer in China [2]. Although the incidence of gastric cancer in young patients is relatively low, it has been increasing worldwide over the past few decades [3, 4].

Data on the clinicopathological features and prognosis of young gastric cancer patients have been both limited and controversial. Compared with middle-aged patients, the proportion of female patients is significantly higher among young patients [5–9]. Furthermore, a significantly higher frequency of histologically undifferentiated tumor types [5, 7, 9] and N3 status [5, 7] are more common in young patients. The prognosis in young gastric cancer patients has been reported to comparable to that in middle-aged patients [5, 6, 8, 9]. On the other hand, Saito et al. [7] reported that prognosis in young gastric cancer patients is worse than that in middle-aged patients. Moreover, little is known about the clinicopathological features and prognosis of gastric cancer in young Chinese patients.

In light of this, we retrospectively analyzed the clinicopathological features and prognosis of 198 young patients and 1096 middle-aged patients with gastric cancer who had been treated with curative surgical resection. The aim of the present study was to assess the clinicopathological features and prognosis of gastric cancer in young patients.

## Methods

### Patients and data

This study was performed in the Xijing Hospital of Digestive Diseases affiliated to the Fourth Military Medical University. From May 2008 to December 2014, a total of 5285 gastric cancer patients underwent treatment in our department. The inclusion criteria were as follows: patients 1. underwent D2 gastrectomy; 2. had no neoadjuvant chemotherapy; and 3. had no distant metastasis. This study was approved by the Ethical Committee of Xijing Hospital, and written consent was obtained from all patients.

**Fig. 1:**
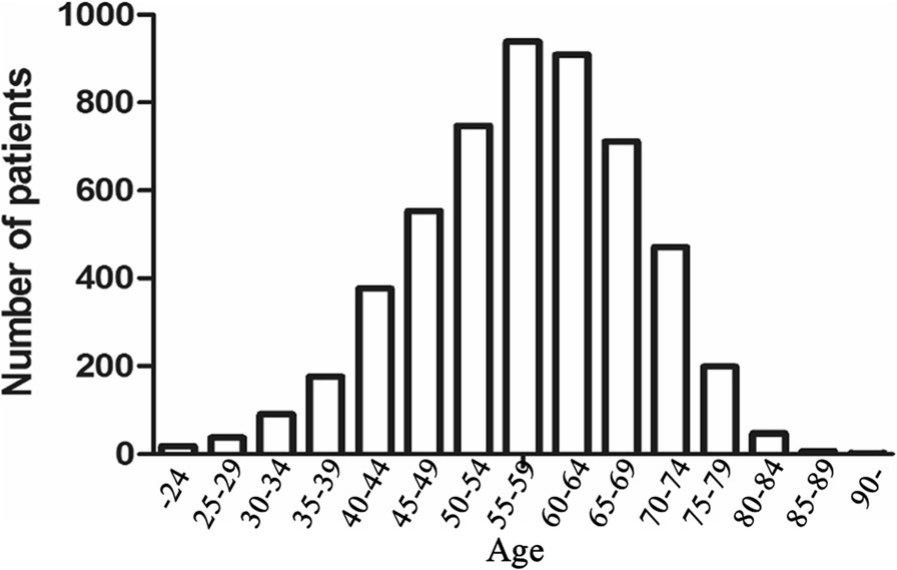
Age histogram for all patients with gastric cancer treated at our institution

In this study, young patients were defined as those aged 40 or under, while middle-aged patients were defined as those within the 10-year range around the median point in the histogram of all patients. The histogram for the 5285 patients with gastric cancer is shown in Fig. 1 and the median point for age was 58 years. Moreover, patients aged 55–64 years in our department, which was the largest part of the histogram, could preferably represent the actual clinicopathological features and prognosis; therefore, the 1096 patients aged 55–64 years were defined as the middle-aged patients.

Among 1294 patients, a curative proximal, distal, or total gastrectomy with a combined standardized lymph node dissection was performed according to the Japanese Classification of Gastric Carcinoma standard. The patients were followed up every 6 months after discharge until December 2015.

Clinicopathological characteristics including age, gender, tumor size, tumor location, histological type, T status, N status, carcinoembryonic antigen (CEA), alpha fetoprotein (AFP) and carbohydrate antigen (CA) 19–9 and CA125 were recorded. The tumors were staged according to the seventh edition of the American Joint Committee on Cancer Tumor Node Metastasis (TNM) classification [10]. A CEA level of 5 μg/L or less, AFP level of 8.1 μg/L or less, CA 19–9 of 27 U/mL or less and CA125 level of 35 μg/L or less were considered to be negative.

### Statistical analysis

Chi-squared test or Fisher’s exact test was used to assess the significant differences in the clinicopathological characteristics. The overall survival (OS) was measured from the time of resection until death or last follow-up. The survival rates were calculated using the Kaplan–Meier method; the relative prognostic importance of the parameters was analyzed with the Cox proportional hazards model. Factors generally considered to be associated with prognosis underwent multivariate analysis. A *P* < 0.05 was considered to be statistically significant.

## Results

The clinicopathological characteristics of 198 young patients and 1096 middle-aged patients are shown in Table 1. Gastric cancer occurred predominantly in women among young patients than that in middle-aged patients (41.9 vs 18.3 %, *P* < 0.001). The proportion of patients with comorbidities was significantly higher in middle-aged patients than that in young patients (24.4 vs 4.5 %, *P* < 0.001). The proportion of tumors located in the upper third was significantly higher in middle-aged patients than that in young patients (36.1 vs 7.6 %), while the proportion of tumors located in the lower third was significantly higher in young patients than that in middle-aged patients (62.1 vs 40.5 %, *P* < 0.001). A tumor size larger than 5 cm was more frequent in middle-aged patients than in young patients (50.6 vs 40.9 %, *P* = 0.012). The proportion of well and moderately differentiated tumors in young patients was significantly lower than that in middle-aged patients (3.5 vs 11.2 %, 8.6 vs 29.5 %); the proportion of poorly differentiated tumors was significantly higher than that in middle-aged patients (82.8 vs 53.6 %, *P* < 0.001). The proportion of T1 tumors in young patients was significantly higher than that in middle-aged patients (25.8 vs 17.5 %), while the proportion of T4 tumors in young patients was significantly lower than that in middle-aged patients (22.2 vs 31.6 %, *P* = 0.012). Rates of CEA, AFP and CA19-9 positivity in young patients were significantly lower than that in middle-aged patients (8.1 vs 22.4 %, 1.5 vs 6.3 %, 13.1 vs 20.2 %, respectively; all *P* < 0.05). The distributions of N status and CA125 were comparable between young and middle-aged patients.

**Table 1:** Clinicopathological features of gastric cancer in young and middle-aged patients

Characteristics	Young	Middle-aged	*P* value
	(*n* = 198)	(*n* = 1096)	
Gender			<0.001
male	115 (58.1)	895 (81.7)	
female	83 (41.9)	201 (18.3)	
Comorbidity			<0.001
Negative	189 (95.5)	829 (75.6)	
Positive	9 (4.5)	267 (24.4)	
Hypertension	0 (0.0)	153 (14.0)	<0.001
Coronary heart disease	0 (0.0)	22 (2.0)	0.064
Diabetes mellitus	2 (1.0)	53 (4.8)	0.014
COPD^a^	0 (0.0)	7 (0.6)	0.389
Brain infarction	0 (0.0)	18 (1.6)	0.094
Chronic hepatitis B	6 (3.0)	12 (1.1)	0.044
Tumor location			<0.001
Upper	15 (7.6)	396 (36.1)	
Middle	58 (29.3)	242 (22.1)	
Lower	123 (62.1)	444 (40.5)	
whole	2 (1.0)	14 (1.3)	
Tumor size			0.012
< 5 cm	117 (59.1)	541 (49.4)	
≥ 5 cm	81 (40.9)	555 (50.6)	
Histologic type			<0.001
Well differentiated	7 (3.5)	123 (11.2)	
Moderately differentiated	17 (8.6)	323 (29.5)	
Poorly differentiated	164 (82.8)	587 (53.6)	
Signet ring cell or mucinous	10 (5.1)	63 (5.7)	
T status			0.012
T1	51 (25.8)	192 (17.5)	
T2	31 (15.7)	168 (15.3)	
T3	72 (36.4)	390 (35.6)	
T4	44 (22.2)	346( 31.6)	
N status			0.067
N0	62 (31.3)	392 (35.8)	
N1	27 (13.6)	205 (18.7)	
N2	41 (20.7)	206 (18.8)	
N3	68 (34.3)	293 (26.7)	
Tumor marker			
CEA			<0.001
Positive	16 (8.1)	245(22.4)	
Negative	182 (91.9)	851 (77.6)	
AFP			0.007
Positive	3 (1.5)	69 (6.3)	
Negative	195 (98.5)	1027 (93.7)	
CA19-9			0.020
Positive	26 (13.1)	221 (20.2)	
Negative	172 (86.9)	875 (79.8)	
CA125			0.152
Positive	14 (7.1)	51 (4.7)	
Negative	184 (92.9)	1045 (95.3)	

^a^ COPD: chronic obstructive pulmonary disease

**Fig. 2:**
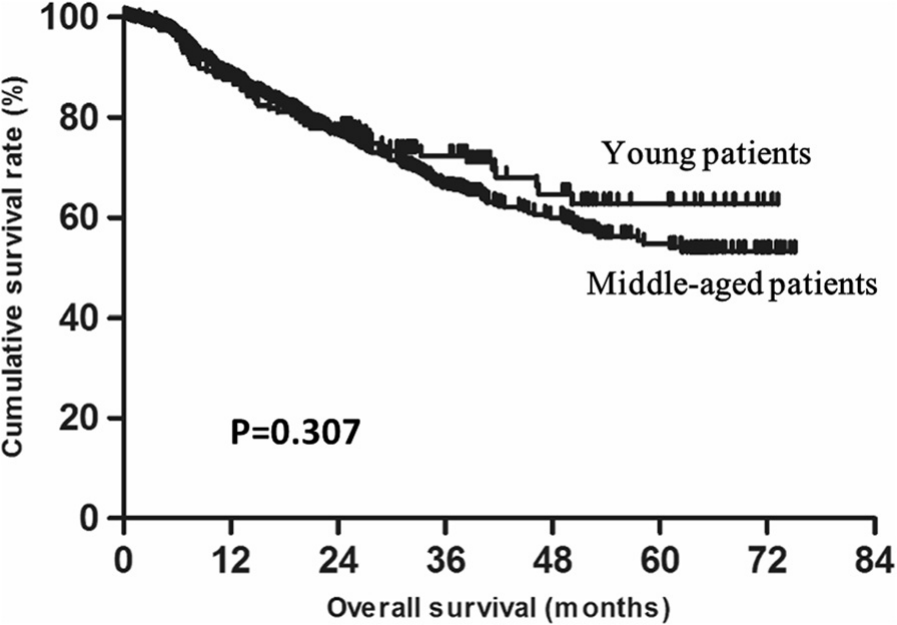
Comparison of overall survival between in young and middle-aged patients

The risk factors for the prognosis of gastric cancer in young and middle-aged patients were analyzed using univariate and multivariate analysis (Table 2). The results indicated that tumor location, tumor size, histological type, T status, N status, CEA, AFP, CA19-9 and CA125 were associated with the prognosis according to the univariate analysis. Tumor size, T status, N status, CEA, CA19-9 and CA125 were independent risk factors for prognosis based on the multivariate analysis. Age was not a risk factor for prognosis in either the univariate or multivariate analysis. The prognosis was analyzed using Kaplan–Meier method. The median overall survival (OS) was 27.28 (1.80–73.13) and 22.90 (1.90–75.10) months in young and middle-aged patients, respectively. The 5-year overall survival rates were comparable between young and middle-aged patients (62.8 vs 54.7 %, *P* = 0.307, Fig. 2).

**Table 2:** Survival analysis of the 1294 gastric cancer patients

Parameter	Univariate Analysis			Mutivariate Analysis		
	β	HR (95 % CI)	*P* value	β	HR (95 % CI)	*P* value
Age (young/older group)	0.152	0.859 (0.642–1.150)	0.307	−0.001	0.999 (0.727–1.372)	0.993
Gender (male/ female)	0.234	1.264 (0.996–1.605)	0.054	0.163	1.177 (0.916–1.513)	0.202
Comorbidity (negative/ positive)	0.078	1.082 (0.824–1.420)	0.572	−0.027	0.973 (0.735–1.288)	0.848
Tumor location (upper/middle/lower third /whole)	−0.139	0.870 (0.774–0.977)	0.019	−0.042	0.958 (0.848–1.083)	0.496
Tumor size (<5 cm/≥5 cm)	1.066	2.905 (2.320–3.637)	0.000	0.238	1.268 (1.014–1.587)	0.038
Depth of invasion (T1/T2/T3/T4)	0.824	2.280 (2.000–2.599)	0.000	0.492	1.636 (1.408–1.901)	0.000
Lymph node metastasis (N0/N1/N2/N3)	0.730	2.075 (1.880–2.290)	0.000	0.505	1.657 (1.482–1.852)	0.000
Histologic type (well/moderately/poorly / signet ring cell or mucinous)	−0.194	0.824 (0.726–0.935)	0.003	−0.050	0.951 (0.840–1.078)	0.436
CEA (negative/ positive)	0.841	2.318 (1.848–2.907)	0.000	0.352	1.422 (1.110–1.822)	0.005
AFP (negative/ positive)	0.640	1.896 (1.325–2.712)	0.000	0.325	1.384 (0.952–2.011)	0.088
CA19-9 (negative/ positive)	0.834	2.303 (1.839–2.882)	0.000	0.365	1.441 (1.137–1.825)	0.002
CA125 (negative/ positive)	1.176	3.242 (2.274–4.622)	0.000	0.729	2.073 (1.442–2.979)	0.000

**Fig. 3:**
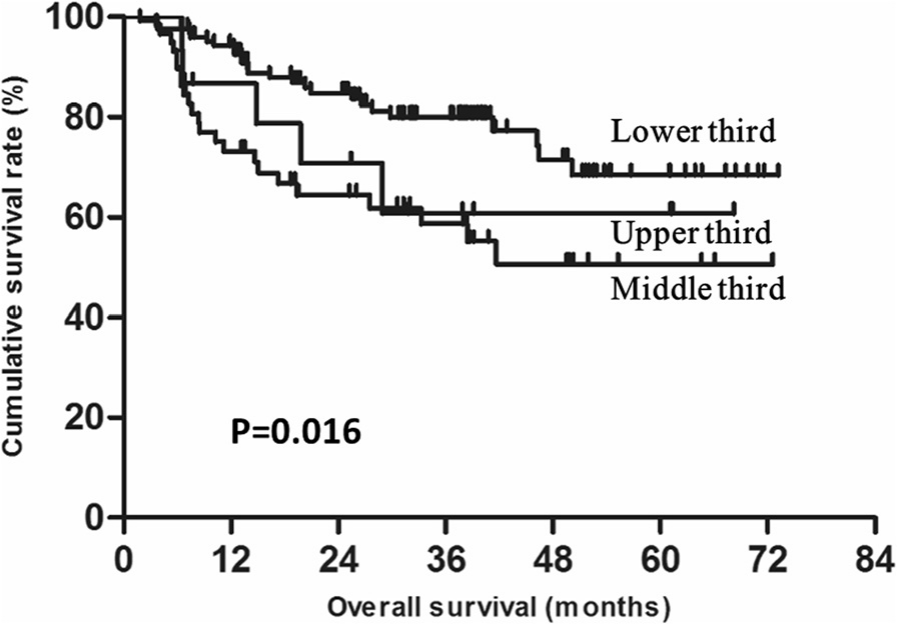
Overall survival in young patients based on tumor location

**Fig. 4:**
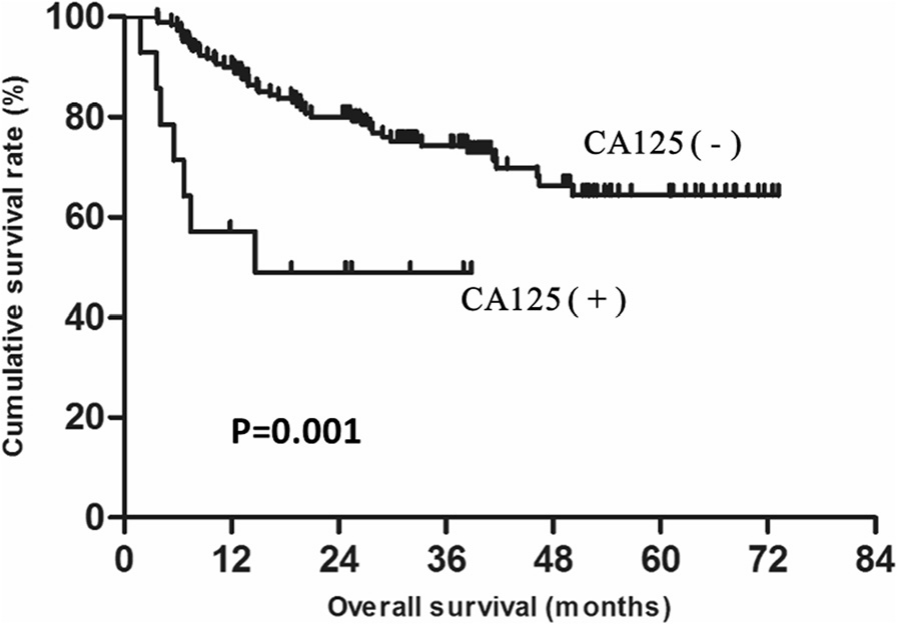
Overall survival in young patients based on level of CA125

To explore the factors impacting prognosis in young gastric cancer patients, univariate and multivariate analyses were performed. The results showed that gender, tumor size, tumor location, T status, N status, CA19-9 and CA125 were associated with prognosis. Moreover, tumor location, T status, N status and CA125 were identified as independent prognostic factors (Table 3). The overall survival of patients with tumors located in the upper or middle third was significantly lower than for those located in the lower third (60.8 % vs 50.6 vs 68.4 %, respectively; *P* = 0.016, Fig. 3). The overall survival of CA125-positive patients was significantly lower than for that of negative patients (49.0 vs 64.4 %, *P* = 0.001, Fig. 4).

**Table 3:** Survival analysis of the 198 young gastric cancer patients

Parameter	Univariate Analysis			Mutivariate Analysis		
	β	HR (95 % CI)	*P* value	β	HR (95 % CI)	*P* value
Gender (male/ female)	0.557	1.746 (1.017–2.997)	0.043	0.015	1.015 (0.572–1.800)	0.959
Comorbidity (negative/ positive)	−3.082	0.046 (0.000–10.360)	0.265	−11.166	0.000 (0.000-NA)	0.973
Tumor location (upper/middle/lower third /whole)	−0.487	0.615 (0.427–0.886)	0.009	−0.407	0.665 (0.428–1.035)	0.048
Tumor size (<5 cm/≥5 cm)	1.063	2.894 (1.651–5.076)	0.000	0.342	1.407(0.737–2.688)	0.301
Depth of invasion (T1/T2/T3/T4)	1.001	2.722 (1.915–3.868)	0.000	0.461	1.586 (1.067–2.358)	0.023
Lymph node metastasis (N0/N1/N2/N3)	1.226	3.406 (2.349–4.939)	0.000	1.066	2.905 (1.932–4.366)	0.000
Histologic type (well/moderately/poorly / signet ring cell or mucinous)	−0.216	0.806 (0.534–1.217)	0.304	−0.052	0.949 (0.638–1.413)	0.797
CEA (negative/ positive)	0.640	1.896 (0.856–4.200)	0.115	0.428	1.534 (0.608–3.871)	0.365
AFP (negative/ positive)	−3.026	0.049 (0.000–913.653)	0.547	−11.184	0.000 (0.000-NA)	0.986
CA19-9 (negative/ positive)	1.036	2.817 (1.505–5.272)	0.001	0.382	1.466 (0.710–3.026)	0.301
CA125 (negative/ positive)	1.260	3.527 (1.578–7.886)	0.002	1.087	2.965 (1.221–7.203)	0.016

## Discussion

The clinical features and prognosis of gastric cancer in young patients remain unclear. In addition, reports of the clinicopathological features and prognosis of young gastric cancer patients in China are rare. We found that the clinicopathological features were significantly different between young and middle-aged patients. However, age was not an independent risk factor for the prognosis of gastric cancer patients in either young or middle-aged patients.

Consistent with previous studies [5–9], the proportion of females in the young patient group was significantly higher than that among middle-aged patients in our study. The reason for this remains unclear. Some investigators suspect that it may be partially due to the higher expression of estrogen receptors in young female patients with gastric cancer [11–13]. Moreover, Kath et al. [14] suggested that the preponderance of female among young patients may be attributed to an association with recent pregnancies. Therefore, estrogen may play a role in the pathogenesis of gastric cancer in young patients; however, this hypothesis requires further investigation [15].

In previous studies [5–7, 9], the proportion of undifferentiated tumors is significantly higher in young patients than that in middle-aged patients. In the current study, there was no significant difference in the proportion of signet ring cell carcinoma and mucinous adenocarcinoma between the two groups. However, the proportion of poorly differentiated tumors was significantly higher in young patients than in middle-aged patients. Some authors attributed these results to the H. pylori infection. Haruma et al. [16] reported that 95 % of the poorly differentiated cases are positive for H. pylori infection in patients younger than 30. In addition, Hirahashi et al. [17] found that the proportion of H. pylori infection is significantly higher in young patients than in middle-aged patients and suggested that H. pylori infection may contribute to the development of poorly differentiated gastric cancer in the young.

Only one report found a significant difference in tumor location between young and middle-aged patients; tumors located in the upper third is more common in middle-aged patients [7]. Our results were consistent with this finding. We know that cardiac cancer arises via cardiac intestinal metaplasia, which is primarily due to gastroesophageal reflux disease [18–20]. In addition, the incidence of gastroesophageal reflux disease increases with age [21, 22], which may result in a higher proportion of upper third tumors in the middle-aged patients.

Previous studies showed that positive rates of CEA, AFP, CA19-9 and CA125 tends to be higher in elderly patients [23–28]. Moreover, we found that higher positive rates of serum CEA, AFP and CA19-9 were also correlated with older age in non-elderly gastric cancer patients. It has been widely reported that higher rates of serum CEA, AFP and CA19-9-positivity in gastric cancer are correlated with larger tumors, higher T status, and upper third tumor [24, 29, 30]. Therefore, higher positive rates of serum CEA, AFP and CA19-9 in the middle-aged patients of our present study may be a result of the higher proportion of larger tumor size, T4 status and upper third tumors in middle-aged group. In contrast to the previous report, the rate of positive CA125 was higher in young patients than in middle-aged patients, although the difference was not significant. It has been reported that higher rates of CA125 positivity are associated with middle and lower third tumors [26, 31]. Therefore, the higher proportion of middle and lower third tumors in young patients in the present study may explain the current results.

In the present study, age was not an independent risk factor for the prognosis of gastric cancer by univariate and multivariate analyses. Most of the previous studies also reported that the prognosis of gastric cancer is comparable between young and middle-aged patients [5, 6, 8, 9]. Only one report showed that the prognosis of the middle-aged patients is significantly better than that of young patients and suggested that age should be a predictor of survival in patients with gastric cancer [7]. This may have been attributed to the relatively small sample size of the latter study.

According to the univariate and multivariable analyses, the independent risk factors for the prognosis of young patients were tumor location, T status, N status and serum CA125. However, the tumor size and histologic type, which are widely known as risk factors for tumor-related death [6, 32, 33], did not demonstrate a correlation with survival in young patients in the present study. This may result from differences in race, sample size, etc. In the present study, better survival was observed in patients with lower third tumors than in those with upper and middle third tumors. Liu et al. [34] also found that the 3-year survival rate is significantly higher in the distal gastric adenocarcinoma group than that in the esophagogastric junctional adenocarcinoma group after R0 resection. It was reported that AFP, CEA and CA19-9 are independent prognostic factors for gastric cancer [27–29, 35]. However, AFP, CEA and CA19-9 did not show prognostic value for young gastric cancer patients in the present study. This may a result of the relatively small number of young gastric patients in the present study.

There are limitations to our study. First, a single hospital-based design might have led to an uncertain amount of selection bias. Second, this was a retrospective analysis; a well-designed randomized clinical trial should be carried to avoid statistical bias in the future. Third, not all of the patients enrolled in our study reached the 5-year follow-up.

## Conclusion

The clinicopathological features were significantly different between young and middle-aged gastric cancer patients, but the prognosis of gastric cancer in young patients was equivalent to that in middle-aged patients. Tumor location, T status, N status and CA125 levels were independent risk factors for the prognosis in young patients.

## Abbreviations

AFP, alpha fetoprotein; CA, carbohydrate antigen; CEA, carcinoembryonic antigen; OS, overall survival

## Additional file

Additional file 1: The power analysis of all statistical analysis. (DOCX 18 kb)
