# Supplementary material for: Clinicopathological features and prognosis of gastric cancer in young patients
Source: BMC Cancer. 2016 Jul 14;16:478. doi: 10.1186/s12885-016-2489-5 (PMC4946107; doi:10.1186/s12885-016-2489-5)
Supplement: Additional file 1: — The power analysis of all statistical analysis. (DOCX 18 kb) [file 12885_2016_2489_MOESM1_ESM.docx]

**Table 1. Clinicopathological features of gastric cancer in young and middle-aged patients**

| Characteristics | Young  (n=198) | Middle-aged  (n=1096) | P value | Power analysis |
| --- | --- | --- | --- | --- |
| Gender |  |  | <0.001 | 1.000 |
| male | 115(58.1) | 895(81.7) |  |  |
| female | 83(41.9) | 201(18.3) |  |  |
| Comorbidity |  |  | <0.001 | 1.000 |
| Negative | 189(95.5) | 829(75.6) |  |  |
| Positive | 9(4.5) | 267(24.4) |  |  |
| Hypertension | 0(0.0) | 153(14.0) | <0.001 |  |
| Coronary heart disease | 0(0.0) | 22(2.0) | 0.064 |  |
| Diabetes mellitus | 2(1.0) | 53(4.8) | 0.014 |  |
| COPD^a^ | 0(0.0) | 7(0.6) | 0.389 |  |
| Brain infarction | 0(0.0) | 18(1.6) | 0.094 |  |
| Chronic hepatitis B | 6(3.0) | 12(1.1) | 0.044 |  |
| Tumor location |  |  | <0.001 | Upper vs Middle: 0.999  Upper vs Lower: 1.000 |
| Upper | 15(7.6) | 396(36.1) |  |  |
| Middle | 58(29.3) | 242(22.1) |  |  |
| Lower | 123(62.1) | 444(40.5) |  |  |
| whole | 2(1.0) | 14(1.3) |  |  |
| Tumor size |  |  | 0.012 | 0.716 |
| <5cm | 117(59.1) | 541(49.4) |  |  |
| ≥5cm | 81(40.9) | 555(50.6) |  |  |
| Histologic type |  |  | <0.001 | Well vs poorly:1.000  Moderately vs poorly differentiated:1.000 |
| Well differentiated | 7(3.5) | 123(11.2) |  |  |
| Moderately differentiated | 17(8.6) | 323(29.5) |  |  |
| Poorly differentiated | 164(82.8) | 587(53.6) |  |  |
| Signet ring cell or mucinous | 10(5.1) | 63(5.7) |  |  |
| T status |  |  | 0.012 | T1 vsT4:0.887 |
| T1 | 51(25.8) | 192(17.5) |  |  |
| T2 | 31(15.7) | 168(15.3) |  |  |
| T3 | 72(36.4) | 390(35.6) |  |  |
| T4 | 44(22.2) | 346(31.6) |  |  |
| N status |  |  | 0.067 |  |
| N0 | 62(31.3) | 392(35.8) |  |  |
| N1 | 27(13.6) | 205(18.7) |  |  |
| N2 | 41(20.7) | 206(18.8) |  |  |
| N3 | 68(34.3) | 293(26.7) |  |  |
| Tumor marker |  |  |  |  |
| CEA |  |  | <0.001 | 1.000 |
| Positive | 16(8.1) | 245(22.4) |  |  |
| Negative | 182(91.9) | 851(77.6) |  |  |
| AFP |  |  | 0.007 | 0.996 |
| Positive | 3(1.5) | 69(6.3) |  |  |
| Negative | 195(98.5) | 1027(93.7) |  |  |
| CA19-9 |  |  | 0.020 | 0.741 |
| Positive | 26(13.1) | 221(20.2) |  |  |
| Negative | 172(86.9) | 875(79.8) |  |  |
| CA125 |  |  | 0.152 |  |
| Positive | 14(7.1) | 51(4.7) |  |  |
| Negative | 184(92.9) | 1045(95.3) |  |  |
| COPD: chronic obstructive pulmonary disease | | | |  |

| **Table 2 Survival analysis of the 1294 gastric cancer patients** | |
| --- | --- |
| Parameter | **Power analysis** |
| Age (young/older group) |  |
| Gender (male/ female) |  |
| Comorbidity (negative/ positive) |  |
| Tumor location (upper/middle/lower third /whole) | upper vs lower:0.897  upper vs whole:0.525  whole vs middle:0.609  whole vs lower:0.712 |
| Tumor size (<5cm/≥5cm) | 1.000 |
| Depth of invasion (T1/T2/T3/T4) | T1vsT2:0.999  T1vsT3:1.000  T1vsT4:1.000  T4vsT2:1.000  T4vsT3:1.000  T2vsT3:0.999 |
| Lymph node metastasis (N0/N1/N2/N3) | N0vsN1:1.000  N0vsN2:1.000  N0vsN3:1.000  N1vsN2:0.997  N1vsN3:1.000  N2vsN3:1.000 |
| Histologic type (well/moderately/poorly / signet ring cell or mucinous) |  |
| CEA (negative/ positive) | 1.000 |
| AFP (negative/ positive) | 0.873 |
| CA19-9 (negative/ positive) | 1.000 |
| CA125 (negative/ positive) | 0.999 |
| **Table 3 Survival analysis of the 198 young gastric cancer patients** | |
| Parameter | Power analysis |
| Gender (male/ female) | 0.639 |
| Comorbidity (negative/ positive) |  |
| Tumor location (upper/middle/lower third /whole) | Middle vs lower:0.882 |
| Tumor size （<5cm/≥5cm） | 0.962 |
| Depth of invasion (T1/T2/T3/T4) | T1vsT2:0.897  T1vsT3:1.000  T1vsT4:1.000  T2vsT4:0.993  T3vsT4:0.917 |
| Lymph node metastasis (N0/N1/N2/N3) | N0vsN2:0.978  N0vsN3:1.000  N1vsN3:1.000  N2vsN3:0.998  N2vsN1:0.744 |
| Histologic type (well/moderately/poorly / signet ring cell or mucinous) |  |
| CEA (negative/ positive) |  |
| AFP (negative/ positive) |  |
| CA19-9 (negative/ positive) | 0.816 |
| CA125 (negative/ positive) | 0.760 |
